# Hypoxic preconditioning enhances the differentiation of bone marrow stromal cells into mature oligodendrocytes via the mTOR/HIF‐1α/VEGF pathway in traumatic brain injury

**DOI:** 10.1002/brb3.1675

**Published:** 2020-05-30

**Authors:** Xiaoyu Yuan, Qianqian Luo, Lihua Shen, Jin Chen, Deqiang Gan, Yechao Sun, Lingzhi Ding, Guohua Wang

**Affiliations:** ^1^ Department of Neurophysiology and Neuropharmacology Institute of Special Environmental Medicine and Co‐innovation Center of Neuroregeneration Nantong University Nantong China; ^2^ Department of Emergency Affiliated Hospital of Nantong University Nantong China; ^3^ Department of Neurology Affiliated Hospital of Nantong University Nantong China

**Keywords:** bone marrow stromal cells, hypoxic preconditioning, oligodendrogenesis, remyelination, traumatic brain injury

## Abstract

**Objective:**

Traumatic brain injury (TBI) results not only in gray matter damage, but also in severe white matter injury (WMI). Previous findings support hypoxic preconditioning (HP) could augment the efficacy of bone marrow stromal cell (BMSC) transplantation in a TBI mouse model. However, whether HP‐treated BMSCs (H‐BMSCs) could overcome remyelination failure after WMI is unclear, and the molecular mechanisms remain to be explored. Here, we focused on the therapeutic benefits of H‐BMSC transplantation for treating WMI, as well as its underlying mechanisms.

**Methods:**

In vitro, BMSCs were incubated at passage 4 in the hypoxic preconditioning (1.0% oxygen) for 8 hr. In vivo, a TBI mouse model was established, and DMEM cell culture medium (control), normal cultured BMSCs (N‐BMSCs), or H‐BMSCs were transplanted to mice 24 hr afterward. Neurobehavioral function, histopathological changes, and oligodendrogenesis were assessed for up to 35 days post‐TBI.

**Results:**

Compared with the control group, improvement of cognitive functions and smaller lesion volumes was observed in the two BMSC‐transplanted groups, especially the H‐BMSC group. H‐BMSC transplantation resulted in a greater number of neural/glial antigen 2 (NG2)–positive and adenomatous polyposis coli (APC)–positive cells than N‐BMSC transplantation in both the corpus callosum and the striatum. In addition, we observed that the expression levels of hypoxia‐inducible factor‐1a (HIF‐1α), phosphorylated mechanistic target of rapamycin (p‐mTOR), and vascular endothelial growth factor (VEGF) were all increased in H‐BMSC–transplanted mice. Furthermore, the mTOR pathway inhibitor rapamycin attenuated the impact of HP both in vivo and in vitro.

**Conclusion:**

The results provided mechanistic evidences suggesting that HP‐treated BMSCs promoted remyelination partly by modulating the pro‐survival mTOR/HIF‐1α/VEGF signaling pathway.

## Graphical abstract

The aim of the present study was to assess the effects of HP‐treated BMSCs (H‐BMSCs) on neuronal behavior and the differentiation of cells in the white matter area following TBI in mice. H‐BMSC transplantation not only ameliorated neurological deficits, but also enhanced axonal regeneration and remyelination in mice after TBI, which may involve the mTOR/HIF‐1α/VEGF pathway. This study provided novel biological and histopathological evidences for TBI treatment that H‐BMSC transplantation may serve as a promising therapeutic option for both gray matter and white matter.

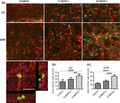

## 1 INTRODUCTION

Bone marrow stromal cells (BMSCs), also known as mesenchymal stem cells, are multipotent nonhematopoietic stem cells (Javazon, Colter, Schwarz, & Prockop, 2001; Li, Ghazanfari, Zacharaki, Lim, & Scheding, 2016). Once extracted, BMSCs can be very proliferative in vitro. After transplantation, BMSCs differentiate into neurons and were reportedly able to facilitate the regeneration of injured axons and promote functional recovery after injuries to the central nervous system (Carbonara et al., 2018; Neirinckx et al., 2015; Shen et al., 2016). However, due to poor survival environment for engrafted cells within the lesion sites restrained the performance of this cell‐based therapy (Wang, Zhou, et al., 2015). Oxygen content in the artery is approximately 12%, whereas under physiological conditions only a concentration of 2%–8% oxygen is available to BMSCs in bone marrow, resulting in poor survival upon transplantation (Mohyeldin, Garzon‐Muvdi, & Quinones‐Hinojosa, 2010). Meanwhile, hypoxic preconditioning (HP) has been reported to increase ischemic tolerance, reduce apoptosis, and cause gene expression alterations (Feng & Bhatt, 2015; Huang et al., 2013). It has been reported in studies on ischemic and hemorrhagic stroke that HP promotes the regenerative capability of the transplanted cells and increases their therapeutic benefits (Chen et al., 2017; Huang et al., 2013).

Traumatic brain injury (TBI) is when the brain is injured by an external force, also known as intracranial injury (Carbonara et al., 2018; Dixon, 2017). Despite varying initial causes of primary injury, TBI as well as ischemic or hemorrhagic stroke shares the common detriment of reduced blood flow and energy failure, resulting in cell death and tissue loss (Shein & Shohami, 2011). HP‐treated BMSC (H‐BMSC) transplantation is regarded as having potential therapeutic benefits for TBI. Chang et al. have reported that HP enhances the secretion of these bioactive factors from the MSCs and the therapeutic potential of the cultured MSC secretome in experimental TBI (Chang et al., 2013). Thus far, there is still a lack of reports about restorative benefits of direct H‐BMSC transplantation for treating TBI.

On the other hand, most preclinical BMSC transplantations in TBI studies greatly emphasize gray matter over white matter (Hu et al., 2019). Actually, TBI results not only in gray matter damage, but also in severe white matter injury (WMI), thereby disrupting signal transmission and eliciting poor functional outcomes (Wang, Shi, et al., 2015). At present, there are no satisfactory therapies to protect TBI patients against either gray matter injury or WMI (Shein & Shohami, 2011; Wang, Shi, et al., 2015). Whether H‐BMSCs could promote remyelination after WMI is unclear, and the mechanisms of BMSCs protecting against TBI‐induced injuries remain to be explored. Therefore, the aim of the present study was to evaluate the effects of H‐BMSCs on neurobehavioral function and the differentiation into oligodendrocytes in the white matter area following TBI in mice. Furthermore, this study aimed to investigate the potential mechanisms involved in this process, potentially providing critical evidence supporting the use of H‐BMSC transplantation for TBI therapy in future clinical trials.

## 2 MATERIALS AND METHODS

### 2.1 Materials and animals

Unless otherwise stated, all chemicals were obtained from Sigma Chemical Company. Goat anti‐mouse or anti‐rabbit IRDye 800CW secondary antibodies were obtained from LI‐COR Biosciences; Alexa Fluor 488 goat anti‐rabbit IgG and Alexa Flour 555 goat anti‐rabbit IgG were obtained from Life Technologies; BCA protein assay kit and RIPA lysis buffer were obtained from Beyotime Institute of Biotechnology.

**FIGURE 1:**
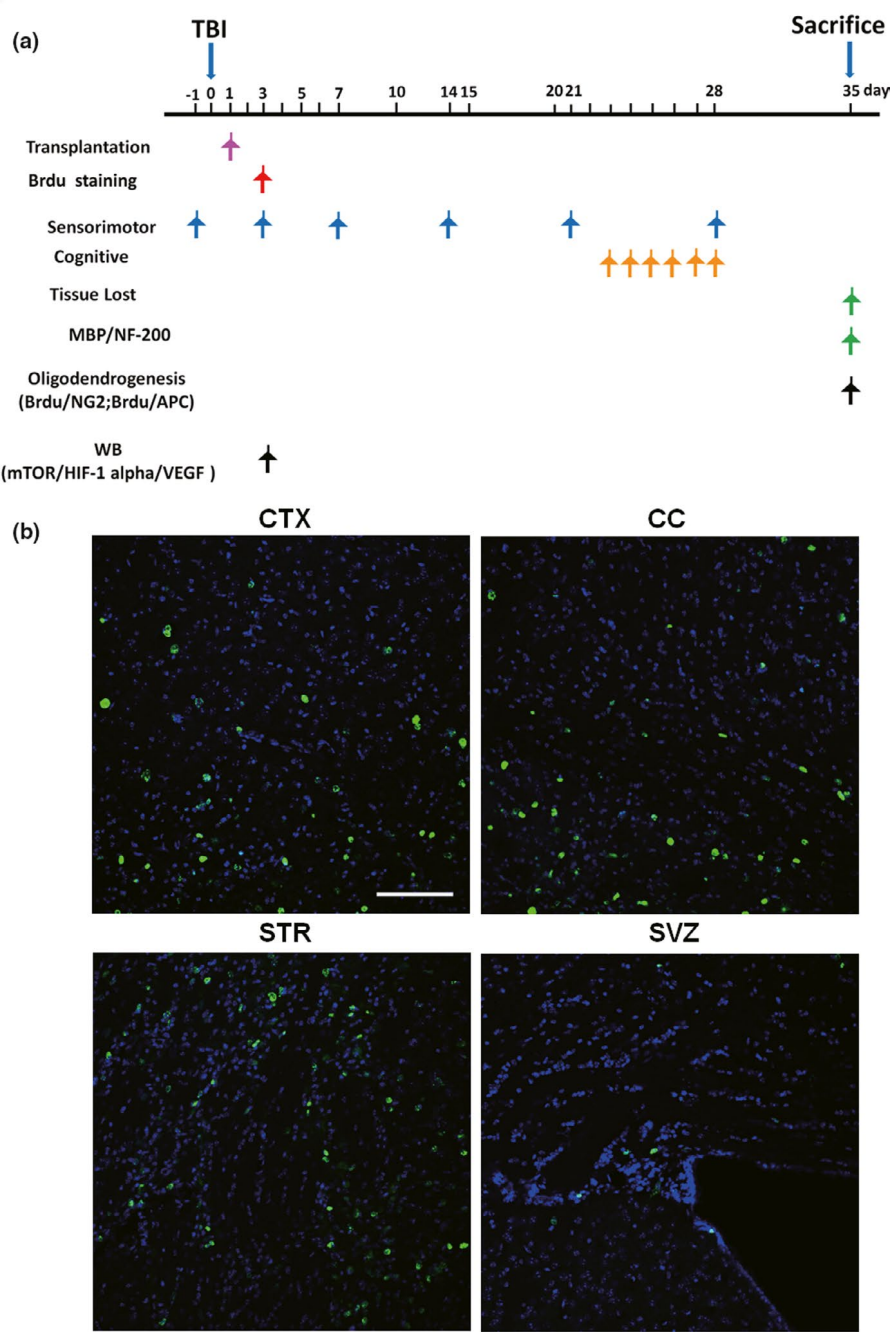
Tracking transplanted cells in the brain after traumatic brain injury (TBI). (a) Temporal schematic of the experimental protocol, the detection times, and the analysis of the different experiments. (b) Visualization of the location of transplanted bone marrow stromal cells (BMSCs) in the corpus callosum (CC) of BMSC‐injected mice. The BMSCs were incubated with 5‐bromo‐2‐deoxyuridine (BrdU) for 3 days before being transplanted into mice by i.v. injection 1 day after TBI. BrdU (green) staining in the cortex (CTX), CC, striatum (STR), and subventricular zone (SVZ) was detected 2 days after BMSC transplantation. These sections were costained with 4'‐6‐diamidino‐2‐phenylindole (DAPI) (blue) to reveal nuclear morphology. Bar indicates 100 μm

The C57BL/6 J male mice (25–30 g body weight) were obtained from the Experimental Animal Center of Nantong University. All animals were housed in stainless‐steel cages under 21 ± 2°C with a relative humidity of 55%–60% and alternating 12‐hr periods of light and dark. After 1 week of acclimatization, the mice were randomly assigned to different groups (*n* = 8 per group). Mice in the control group were subjected to the same procedures as other groups but without fasting. On the day of animal sacrifice, mice were anesthetized with 1% pentobarbital sodium (40 mg/kg body weight, i.p.), decapitated, and received myocardial perfusion with phosphate‐buffered saline (PBS). Mouse brains were removed and processed for total RNA extraction and protein concentration determination (Luo et al., 2018). All procedures of animal housing, surgery, sacrifice, and sample taking were conducted in accordance with the Animal Management Rules of the Ministry of Health of China with ethical approval from the Animal Ethics Committees of Nantong University: Reporting In Vivo Experiments (ARRIVE) guidelines (https://www.nc3rs.org.uk/arrive‐guidelines). The animals were tested for behavior tests or sacrificed as in Figure 1a.

### 2.2 BMSC isolation and culture

Bone marrow stromal cells isolation was conducted as previously described (Deng et al., 2004). The tibias and femurs of C57BL/6 J mice at 4–6 weeks of age were used to extract bone marrow, which was flushed from the bone cavity into an Eppendorf tube with low‐glucose Dulbecco's modified Eagle medium (L‐DMEM, Invitrogen) supplemented with 10% fetal bovine serum (FBS, Invitrogen). After 5 min of centrifuging at 200 *g* for 5 min, cells in the bone marrow suspension were cultured under 37°C in a standard humidified incubator with 95% air/5% CO_2_. Three days after the cells seeded, culture medium was removed and replaced with fresh medium. Seeded cells were harvested at the 4th passage and then injected into mice via intravenous (i.v.) injection. Some batches of BMSCs were labeled with 30 μg/ml bromodeoxyuridine (5‐bromo‐2‐deoxyuridine, BrdU; Sigma) 3 days before intravenous administration of BMSCs to assess cell migration in the TBI area (Cui & Almazan, 2007). After digestion and centrifugation, the live cells were resuspended in 1 ml of L‐DMEM containing 2 × 10^6^ cells for transplantation. The remaining unlabeled cells were incubated in flasks under 37°C to investigate the effects of HP on the mechanistic target of rapamycin (mTOR) signaling pathway.

### 2.3 Hypoxic preconditioning of BMSCs

Taken together with previously reported findings, BMSCs were incubated at passage 4 in the hypoxic preconditioning (1.0% O_2_) for 8 hr (Chen et al., 2017; Huang et al., 2013; Sun et al., 2015). Cultured BMSCs were maintained under either normoxic conditions (21% O_2_) or in a fine C‐Chamber Hypoxia Chamber (BioSpherix) in which the oxygen content was fixed at 1% with a residual gas mixture composed of 5% carbon dioxide balanced with nitrogen. For in vitro rapamycin (RAPA) treatment, RAPA (Sigma‐Aldrich, R0395) was dissolved in DMSO (Sigma‐Aldrich, D2650) and was added to BMSCs immediately at a concentration of 5 μg/ml before hypoxic treatment. DMSO alone was used as a vehicle control.

### 2.4 Induction of TBI and experimental groups

Traumatic brain injury was induced according to a previously described method (Wang et al., 2013). Three percent isoflurane in a 67% N_2_O/30% O_2_ mixture (induction) was used for mouse anesthesia. Once the mice were no longer responding to tail pinches, they received 1.5% isoflurane from a nose cone to maintain the anesthetic effects. Each contusion injury was created using a pneumatically driven controlled cortical impact (CCI) device (Precision Systems and Instrumentation) with a 3‐mm flat‐tip impounder (velocity, 3.5 m/s; duration, 150 ms; depth, 1.5 mm). As soon as injury was induced, the bone flap was replaced and sealed with Koldmount cement (Vernon Benshoff), and the mouse scalp was sutured. Sham animals were subjected to all procedures in the protocol (i.e., surgery, anesthesia, craniotomy, and recovery) but not CCI. Only a few mice (<2%) were excluded due to lost ability of independent eating and drinking, signs of pain, or infections at the suture wound, etc. The time points for the assessment of different parameters are illustrated in Figure 1a.

The cell transplantation treatment after TBI was performed in mice under aseptic conditions as previously described (Chen et al., 2017). Twenty four hours after TBI induction, each mouse in the vehicle‐treated group (control) received an intravenous injection of DMEM (1 ml) via its tail, while mice in the normal BMSC (N‐BMSC)–treated group and the H‐BMSC–treated group were administrated with N‐BMSCs (2 × 10^6^) and H‐BMSCs (2 × 10^6^). On the day of animal sacrifice, all mice were anesthetized with an intraperitoneal injection of overdosed chloral hydrate (400 mg/kg) and sacrificed 3 or 35 days after the operation for the identification of engrafted BMSCs. BrdU powder (ab6326, Abcam) was dissolved in saline and injected into the mice at a concentration of 50 mg/kg/time via intraperitoneal (i.p.) injection for 7 days (twice a day, intervals of at least 8 hr) beginning on day 3 after TBI.

### 2.5 Measurements of brain injury volume

Photographs of the brain slices were taken with a Leica camera (DM5000B, Leica) for the measurement of lesion volumes using the ImageJ analysis software (National Institutes of Health). The percentage lesion volume was calculated using the formula ((*V*_C_ − *V*_L_)/*V*_C_) × 100 by an investigator blinded to the study groups. *V*_L_ = volume of uninjured tissue in the ipsilateral hemisphere subjected to CCI (right side), while *V*_C_ = volume of the contralateral hemisphere (left side).

### 2.6 Wire‐hanging test

The wire‐hanging test apparatus is comprised of a 50‐cm‐long 2‐mm diameter stainless‐steel bar placed on two vertical supports 37 cm above a flat surface. Mice were placed on the bar in the middle between two supports and were observed for 30 s with a total of four repeats. The amount of time spent hanging was recorded, and each mice was scored 0–4 according to its performance: 0, the mouse fell off the bar; 1, the mouse used 2 forepaws to hang on the bar; 2, the mouse hung onto the bar while also attempted to climb onto the bar; 3, the mouse hung onto the bar using not only 2 forepaws but also 1 or both hind paws; 4, the mouse hung onto the bar with all 4 paws and tail wrapped around the bar; and 5, the mouse was able to escape to either of the supports.

### 2.7 Cylinder test

The adapted cylinder test for mice was conducted to assess their function of forelimb use and rotation asymmetry. Each mouse was placed in a 15‐cm‐tall and 9‐cm‐diameter transparent cylinder, and its performance was videotaped. In order to videotape the forelimb movements of the mice, a mirror was placed behind the cylinder with a specific angle to provide a view for observation. The following criteria were used to classify the status of forelimb use during the mouse's first wall touch after rearing and during lateral exploration: (a) “independent wall placement,” where a first forelimb touched the wall during a full rear; (b) “both movement,” where both forelimbs were used simultaneously during a full rear and for lateral movements along the wall; (c) “left/right forelimb‐independent movement” and “both movement,” if the left/right forelimb was placed on the wall while the other forelimb was then placed on the wall without the first forelimb losing touch with the wall; notably, only one “both” movement should be recorded along with the “left/right forelimb‐independent movement” even if the other forelimb touched the wall several times; and (d) “both movement” if the mouse used both forelimbs in an alternating manner to move along the wall laterally.

### 2.8 Grid‐walking test

The grid‐walking test is a sensitive test for evaluating sensorimotor coordination following TBI. Each mouse was examined in a clear Plexiglas chamber consisting of a 1‐m long walkway with irregularly spaced metal rungs, and each mouse was required to traverse over the rungs to reach the end. If the hind limb failed to grasp a bar and the mouse fell between the bars, it was considered a footfall error, and the total number of errors was recorded. Each mouse was demanded to walk over the grid three times, and the whole process was videotaped. The footfalls were counted by a blind observer watching the videos, and the average number of footfall errors was calculated.

### 2.9 Morris water maze

The Morris water maze (MWM) test was conducted in accordance with previously described procedures (Vorhees & Williams, 2006). The time course of the Morris water maze is shown in Figure 1a. To adapt to the water maze, animals were moved into the behavior room two days before surgery. The water maze was comprised of a black circular water tank (120 cm in diameter, 38 cm tall, and 30 cm deep) filled with room temperature water (25 ± 1°C), with a hidden platform 1 cm below the water surface. The pool was divided into four quadrants, and the platform was placed at the midpoint of one quadrant. After surgery, each mouse received training every day for four consecutive days. Before the first training session, each mouse was placed on the platform for 15 s, allowed to swim for 30 s, and was manually put back onto the platform for another 15‐s resting period. For each trial, the mouse was placed in a different quadrant. A digital camera and the ANY‐maze software (Stoelting Co.) were used to record and calculate the escape latency (time required to find the hidden platform) and swimming distance from starting point to the platform. If a mouse failed to locate the platform within 1 min, the escape latency was recorded as 60 s, while the mouse was manually put onto the platform and stayed for 10 s afterward. The platform was removed on the tenth day after surgery. Each mouse was put into the quadrant where the platform used to be (target quadrant), and the time duration of the mouse remaining in this quadrant within one minute was recorded. Each animal was dried with a towel and kept warm after each test.

### 2.10 Immunofluorescence staining

For histopathological examinations, each mouse was anesthetized and received myocardial perfusion with normal saline followed by 4% paraformaldehyde solution. Mouse brains were postfixed in 4% paraformaldehyde solution for another 24 hr and dehydrated for 2 days in a gradient of 20% and 30% sucrose in 0.1 M PBS under 4°C. The brains were then sliced into 25‐μm‐thick free‐floating sections using a cryostat device and incubated with 4% goat serum to reduce nonspecific binding. Brain sections were subsequently incubated overnight under 4°C with rabbit anti‐myelin basic protein (MBP) antibody (1:200, Abcam), mouse anti‐hypophosphorylated neurofilament H (NF200) (1:100, Abcam), mouse anti‐BrdU (5‐bromo‐20‐deoxyuridine, 1:800, Sigma‐Aldrich), mouse anti‐adenomatous polyposis coli (APC, also known as CC‐1; 1:50, Abcam), and mouse anti‐neural/glial antigen 2 (NG2; 1:200, MAB5384, Millipore). The sections were washed with 0.01 M PBS three times and incubated under room temperature in DyLight 594 or Alexa Fluor 488 goat anti‐rabbit/mouse IgG secondary antibody (Jackson ImmunoResearch Laboratories, Inc.) for 1 hr. Then, the sections were stained with Hoechst 33,342 (5 mg/ml) at 37°C for 15 min, followed by mounting using mounting medium. All images were processed for cell counting as previous method (Chen et al., 2017). Briefly, five slides from each brain (Bregma + 1, +0.5, 0, −0.5, −1 mm), with each slide containing eight fields from cortex and striatum of IBZ were digitized under a 20 × objective using a Leica DM4000B fluorescence microscope, a CCD (charge‐coupled device) camera, and Leica Qwin software. Actually, mean cell counts were calculated from eight random microscopic fields at 200 magnification of each section, respectively, and three consecutive sections were analyzed for each animal. The confocal fluorescence images were collected using a Leica TCS SP8 system with the following parameters: 40 × objective, 1,024 × 1,024 format, 200 Hz speed, bidirectional X, 1 × zoom, 900 gain, and 0 background. All fluorescence values were generated and analyzed semi‐quantitatively with ImageJ software (National Institutes of Health). Counts and fluorescence quantification were made by an investigator who was blind to experimental group assignment.

### 2.11 Western blot analysis

After saline perfusion, tissues around the injection‐side corpus callosum (CC) were collected, washed with PBS, homogenized, and sonicated. The BCA protein assay was performed to measure protein concentration of each sample. Aliquots of the extract containing approximately 30 μg of protein were loaded and run on a 10% separating SDS‐PAGE gel under reducing conditions, and the proteins were subsequently transferred to a membrane by Bio‐Rad system. The blots were blocked in 5% nonfat milk, washed with PBS, and incubated with primary antibodies under 4°C overnight, including rabbit anti‐vascular endothelial growth factor (VEGF; 1:1,000, Abcam), rabbit anti‐phosphorylated p‐mTOR (Ser2488), mTOR, hypoxia‐inducible factor‐1a (HIF‐1α; 1:1,000, Cell Signaling Technology), and β‐actin (1:10,000, A2228, Sigma). Blots were then incubated under room temperature for 2 hr with goat anti‐mouse or anti‐rabbit IRDye 800CW secondary antibody (1:5,000). Band intensities were measured by using an Odyssey infrared imaging system (LI‐COR Biosciences).

### 2.12 Statistical analysis

The GraphPad Prism 5.0 software was used for statistical analysis. Variance homogeneity examination suggested that the data did not substantially deviate from normality. All values were expressed as mean ± *SD*. Significant differences between groups were evaluated with one‐way analysis of variance (ANOVA) and Tukey's post hoc tests for multiple comparisons. The behavioral test data were analyzed with two‐way repeated‐measures ANOVA and Tukey's post hoc tests. A value of *p* < .05 was considered statistically significant.

## 3 RESULTS

### 3.1 Tracking transplanted cells in the brain after TBI

To determine whether BMSCs reached the brain via veins, animals were first injected with BMSCs, which were marked with BrdU advance, via i.v. injection. BrdU fluorescence in the regions of the corpus callosum (CC), subventricular zone (SVZ), cortex (CTX), and striatum (STR) was examined by immunofluorescence (Figure 1b). Two days after administration of BMSCs, mouse brains were obtained, sliced, and processed with BrdU and Hoechst staining procedures. Figure 1b shows that BrdU was found in all of the above regions in the brain 2 days after BMSC transplantation indicating that exogenous BMSCs that were transplanted 2 days prior reached the brain, particularly the CC, CTX, and STR (Figure 1b).

### 3.2 BMSC transplantation ameliorated tissue loss and sensorimotor deficits

**FIGURE 2:**
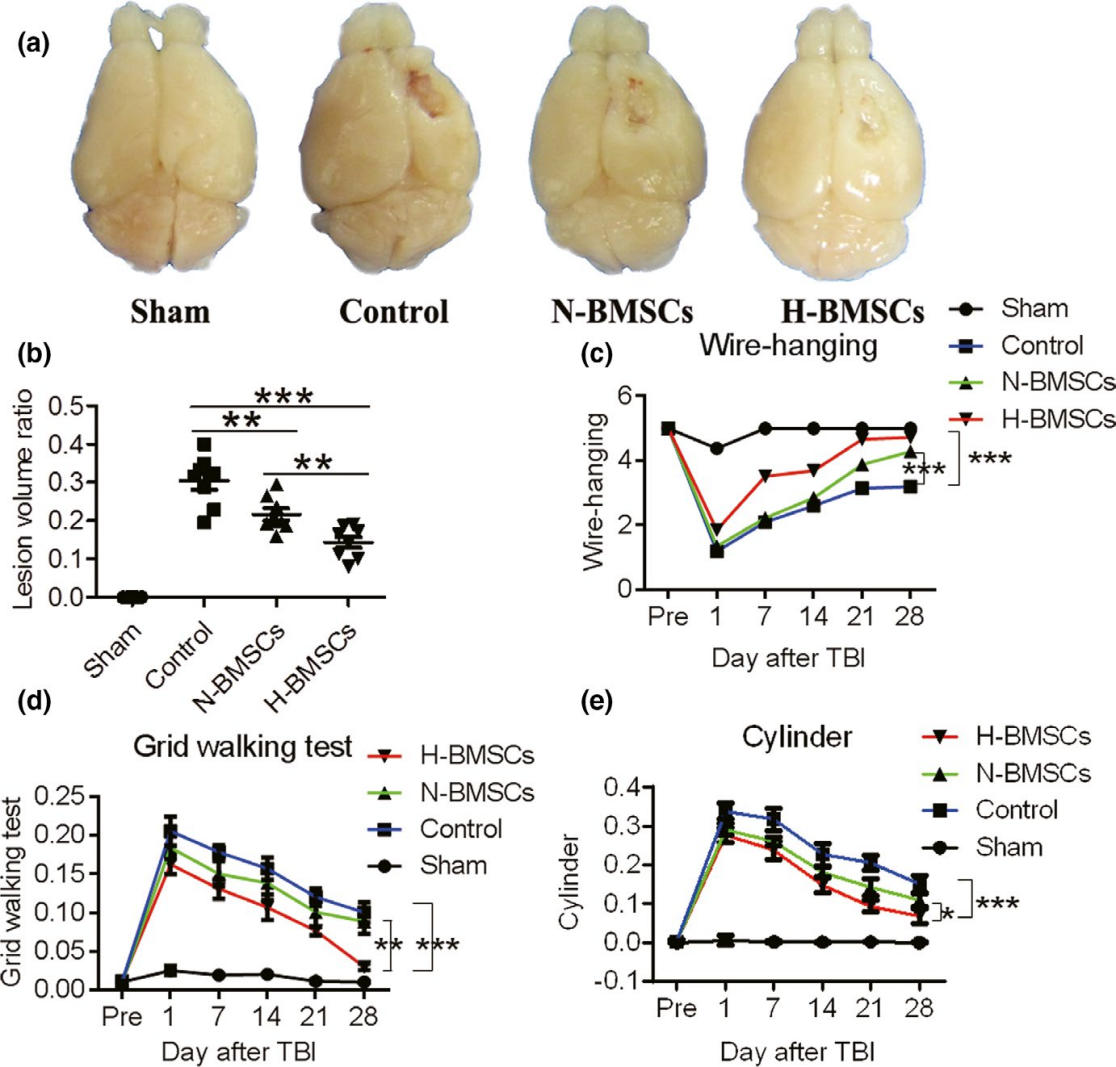
BMSC transplantation conferred protection against tissue loss and long‐term motor deficits due to controlled cortical impact (CCI) injury. (a) The groups transplanted with BMSCs exhibited significantly less tissue loss (cavity size) produced by CCI trauma 35 days after TBI than did the control group. (b) Quantitation of the differences in the lesion volumes 35 days postinjury between the BMSC‐treated and untreated mice (*n* = 8). (c) The wire‐hanging test: BMSC treatment attenuated motor deficits leading to higher performance scores than those in the control group for as many as 35 days postinjury (*n* = 4). (d) Grid test: The administration of BMSCs also diminished biased forelimb use for as many as 4 weeks postinjury (*n* = 8). (e) Cylinder test: The administration of BMSCs also diminished biased forelimb use for as many as 35 days postinjury (*n* = 4). Data are all presented as the mean ± *SD*. *p < .05, **p < .01

To investigate whether BMSC treatment is beneficial to TBI, we first established a TBI mouse model. In addition, we examined whether BMSC injection could improve motor and cognitive deficits in the TBI model. Cortical damage was induced by CCI as expected, with a cavity visible to the naked eye even after 35 days in the control animals. In comparison with the control animals, animals transplanted with BMSCs, particularly H‐BMSC–treated animals, exhibited a smaller cavity (Figure 2a and b). As was expected, TBI caused worse motor deficits in the control group in comparison with those in the sham group in terms of lower wire‐hanging test scores. The grid‐walking test and cylinder test also indicated that BMSC transplantation exhibited protective effects against TBI‐induced motor deficits post‐TBI. Results suggested that TBI‐induced motor deficits were ameliorated by BMSC treatment in a time‐dependent manner, particularly within 7 days after TBI in the H‐BMSC treatment group (Figure 2c–e).

### 3.3 H‐BMSC transplantation improved cognitive function

**FIGURE 3:**
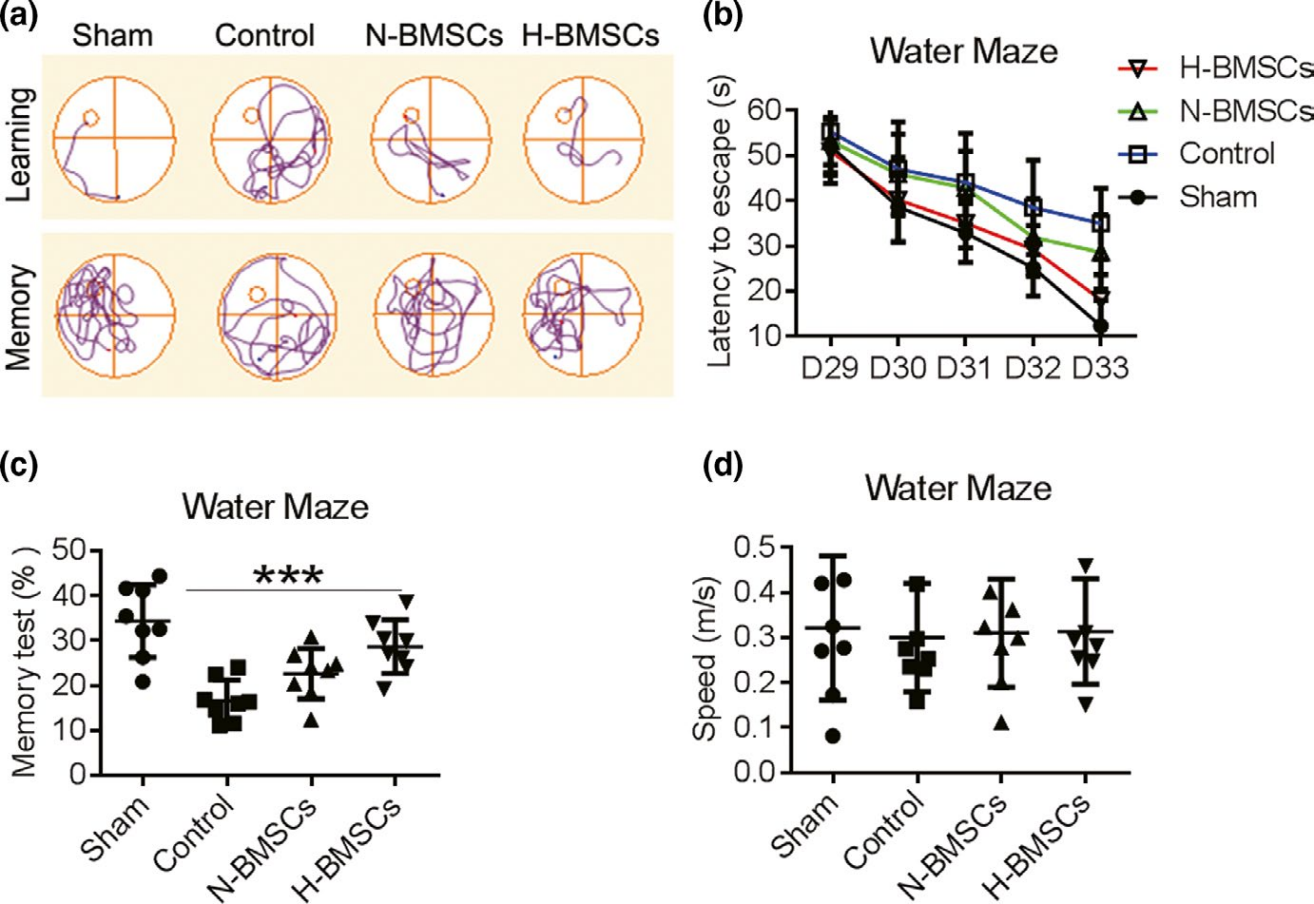
BMSC transplantation improved cognitive function. (a) Cued learning response: a typical swim pattern of mice from the sham, control, normal cultured BMSC (N‐BMSC)–treated, and hypoxia preconditioning–treated BMSC (H‐BMSC)–treated groups during the localization of the platform within the Morris water maze. Cued memory response: a typical swim pattern of mice from the sham, control, N‐BMSC–treated, and H‐BMSC–treated groups during attempts to locate the platform within the Morris water maze based on memory. (b) Time required for mice from these four groups to locate the platform during the cued learning response 29–33 days postinjury (*n* = 8). (c) The percentage of time spent in the same quadrant as the platform after training from 23 to 27 days for mice from the sham, control, N‐BMSC–treated, and H‐BMSC–treated groups (*n* = 8). (d) The mean swimming speed on day 28 (*n* = 8). Data are all presented as the mean ± *SD*. *p < .05 versus control and #*p* < .05 versus N‐BMSCs

Unlike motor deficits, changes in cognitive functions appeared at least 28 days post‐TBI as determined via the MWM test (Figure 3). The mice in the control group showed worse function of memory recall and required longer time to locate the underwater platform than the sham mice. However, mice treated with BMSCs were able to stay longer in the same quadrant as the platform and were faster at locating the platform than the control mice (Figure 3a). The time needed for the mice in the control group was longer than 55 s to locate the platform even after 29 and 33 days post‐TBI. On the other hand, N‐BMSC treatment shortened the time required by the mice to locate the platform from 53 to 28 s, while H‐BMSC treatment shortened the time from 50 to 18 s (Figure 3b). In addition, significant improvements in spatial memory test performances were observed after treatment with BMSCs, particularly with H‐BMSCs (Figure 3c). However, no differences were observed in swimming speeds between the control and BMSC‐transplanted mice (Figure 3d).

### 3.4 H‐BMSC transplantation attenuated neurofilament impairment and demyelination in the brain after TBI

**FIGURE 4:**
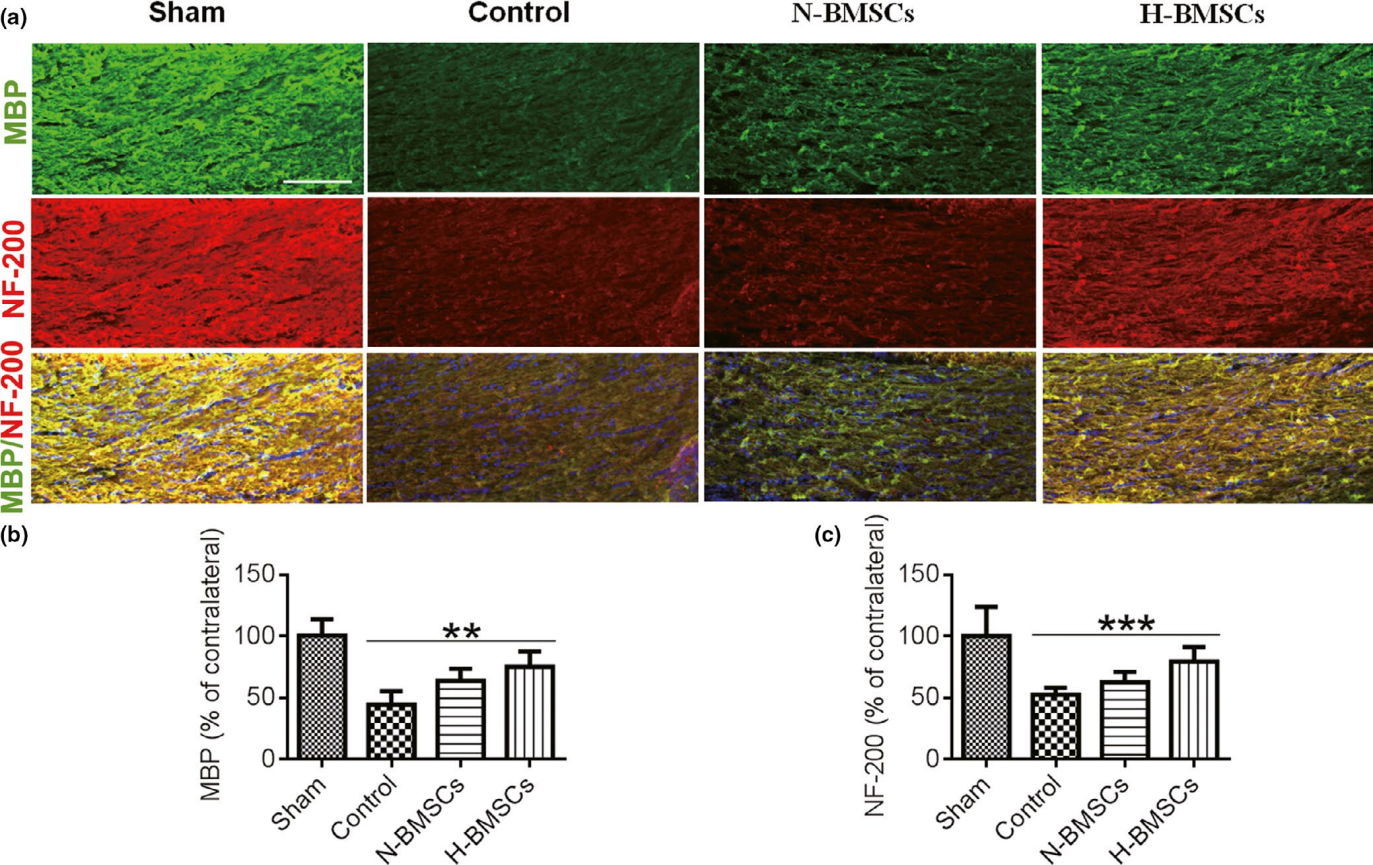
Changes in CC remyelination following transplantation of BMSCs. (a) Representative confocal images showing myelin basic protein (MBP; green) and hypophosphorylated neurofilament H (NF200; red) immunofluorescence. The scale bar represents 50 μm. (b) Pooled data showing the fluorescence intensity of MBP in the CC 35 days after TBI. One‐way ANOVA showed a significant effect of H‐BMSC compared with the control treatment (*n* = 6 mice). (c) Pooled data showing the fluorescence intensity of NF200 in the CC 35 days after TBI. One‐way ANOVA showed a significant effect of H‐BMSC compared with the control treatment (*n* = 6 mice). These sections were costained with DAPI (blue) to reveal nuclear morphology. Data are all presented as the mean ± *SD*. **p < .01, ***p < .001

To determine whether BMSC injections influenced demyelination and neurofilaments in the CC after TBI, we detected the expression of markers for NF200 and MBP via immunohistochemistry of the brain slices of the CC (Figure 4). Neurofilaments are a class of intermediate filaments that are found in neurons. These filaments form the structure of the cytoskeleton and are particularly abundant in axons. The expression of MBP and NF200 in the CC 35 days after TBI was lower in the control group than in the sham group, indicating a reduction in the number of myelinated axons in the group exposed to TBI. In addition, demyelination and neurofilament impairment were observed in the CC after TBI (Figure 4a–c). However, the expression levels of MBP and NF200 were higher in the BMSC‐transplanted groups than in the control group. Notably, the repair was more efficient with H‐BMSC treatment than with N‐BMSC treatment. These results demonstrated that H‐BMSC transplantation could induce remyelination and neurofilament regeneration in the CC 35 days after TBI.

### 3.5 HP enhanced BMSC differentiation after transplantation in TBI

**FIGURE 5:**
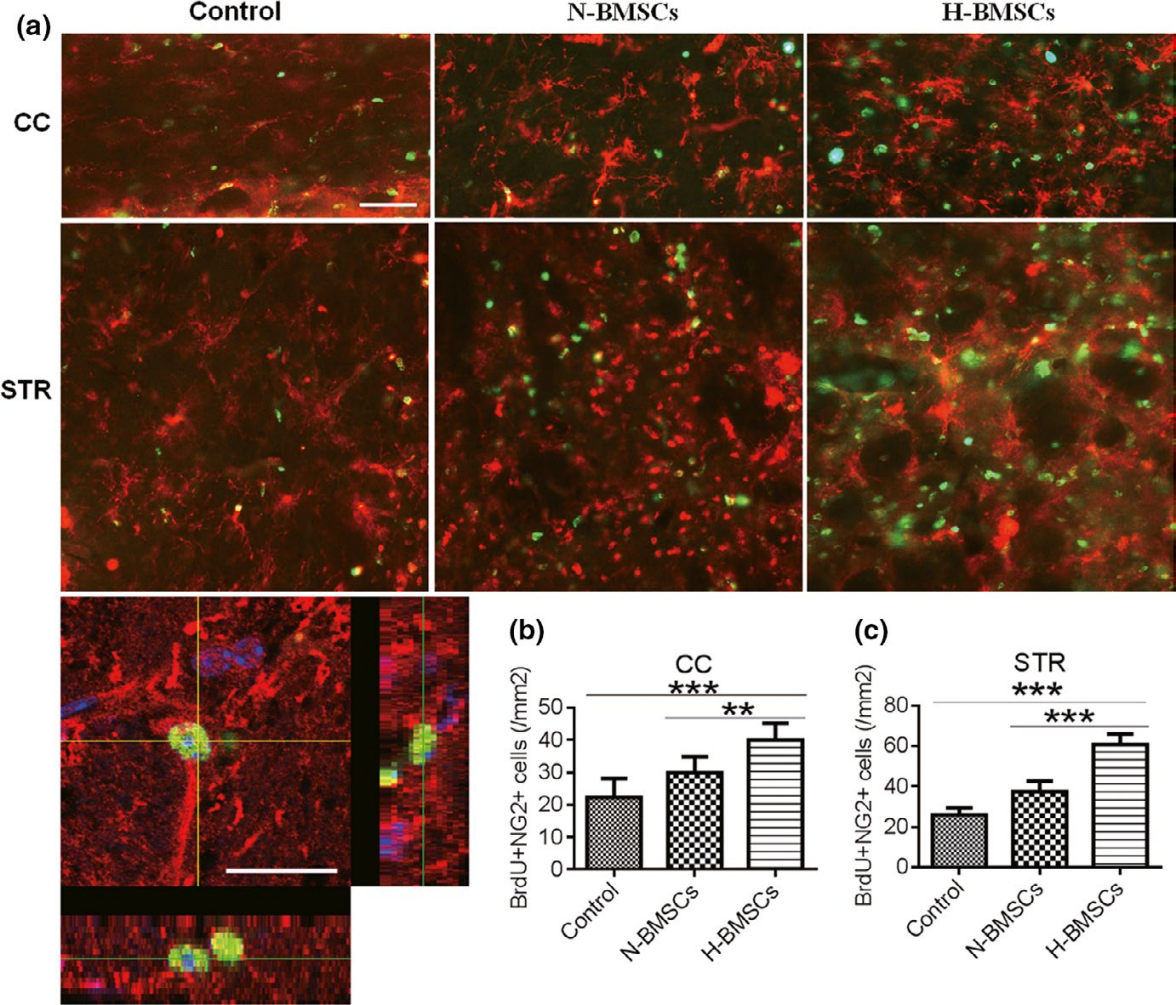
H‐BMSCs improved the differentiation of BMSCs in the CC and STR of TBI mouse brains. (a) The CC and STR in the brain 35 days after TBI were double‐labeled with antibodies against neural/glial antigen 2 (NG2; red) and BrdU (green). The overlay images are shown. The scale bars represent 100 μm. (b) Pooled data showing the effect of BMSC treatment on the number of NG2^+^/BrdU‐positive cells in the CC 35 days after TBI. One‐way ANOVA showed a significant effect of H‐BMSC compared with the control treatment (*n* = 6 mice). (c) Pooled data showing the effect of BMSC treatment on the number of NG2^+^/BrdU‐positive cells in the STR area at day 35 after TBI. One‐way ANOVA showed a significant effect of H‐BMSC compared with the control treatment (*n* = 6 mice). These sections were costained with DAPI (blue) to reveal nuclear morphology. Data are all presented as the mean ± *SD*. **p < .01, ***p < .001

**FIGURE 6:**
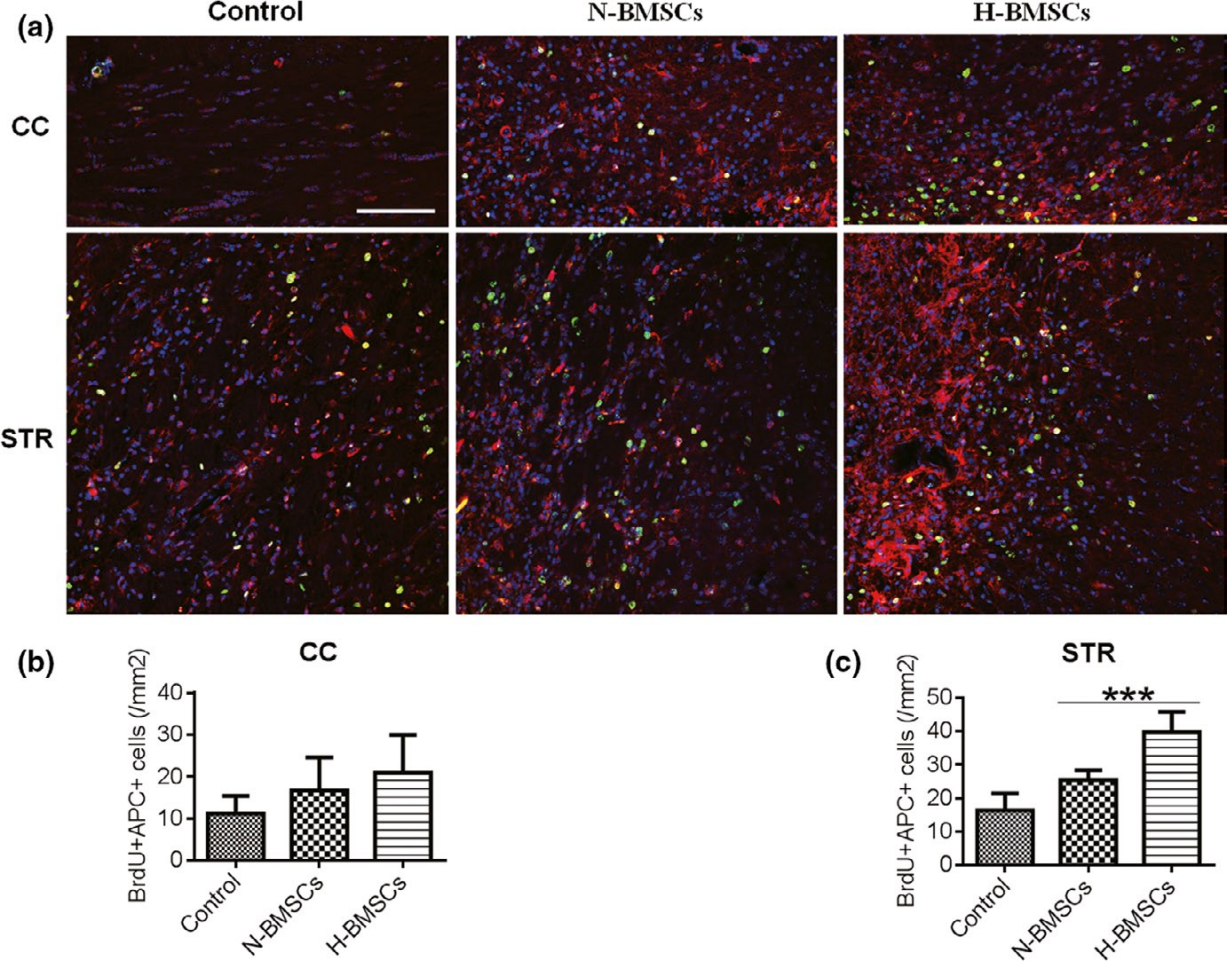
H‐BMSCs improved the differentiation of BMSCs into mature oligodendrocytes in the CC and STR of TBI mouse brains. (a) The CC and STR in the brain 35 days after TBI were double‐labeled with antibodies against adenomatous polyposis coli (APC; red) and BrdU (green). The overlay images are shown. The scale bars represent 100 μm. (b) Pooled data showing the effect of BMSC treatment on the number of APC^+^/BrdU‐positive cells in the CC 35 days after TBI. One‐way ANOVA showed a significant effect of H‐BMSC compared with the control treatment (*n* = 6 mice). (c) Pooled data showing the effect of BMSC treatment on the number of APC^+^/BrdU‐positive cells in the STR area at day 35 after TBI. One‐way ANOVA showed a significant effect of H‐BMSC compared with the control treatment (*n* = 6 mice). These sections were costained with DAPI (blue) to reveal nuclear morphology. Data are all presented as the mean ± *SD*. **p < .01, ***p < .001

BrdU‐positive cells (green) represented engrafted and proliferative BMSCs in the CC. NG2 is a rat integral membrane proteoglycan located on the plasma membrane of various types of cells, such as the oligodendrocyte progenitor cells (OPCs) and other progenitor cell populations (Goncalves et al., 2018). Using APC protein as a marker of mature oligodendrocytes, double immunostaining for BrdU/NG2 (Figure 5) and BrdU/APC (Figure 6) was used to assess BMSC differentiation and proliferation, respectively, both in the CC and STR. Observation of each type of cells indicated advanced differentiation and neurogenesis in the TBI brain after BMSC transplantation. In addition, all the BrdU‐positive cells were colocalized with NG2, suggesting that BrdU was mainly located in OPCs. Furthermore, enhanced regeneration was observed in the H‐BMSC–treated group compared with that in the N‐BMSC–treated group both in the CC and the STR (Figures 5 and 6), indicating that the protection induced by H‐BMSCs was mediated through the induction of the differentiation of OPCs from BMSCs.

### 3.6 H‐BMSC transplantation prevented the inhibition of the mTOR pathway in the brain after TBI

**FIGURE 7:**
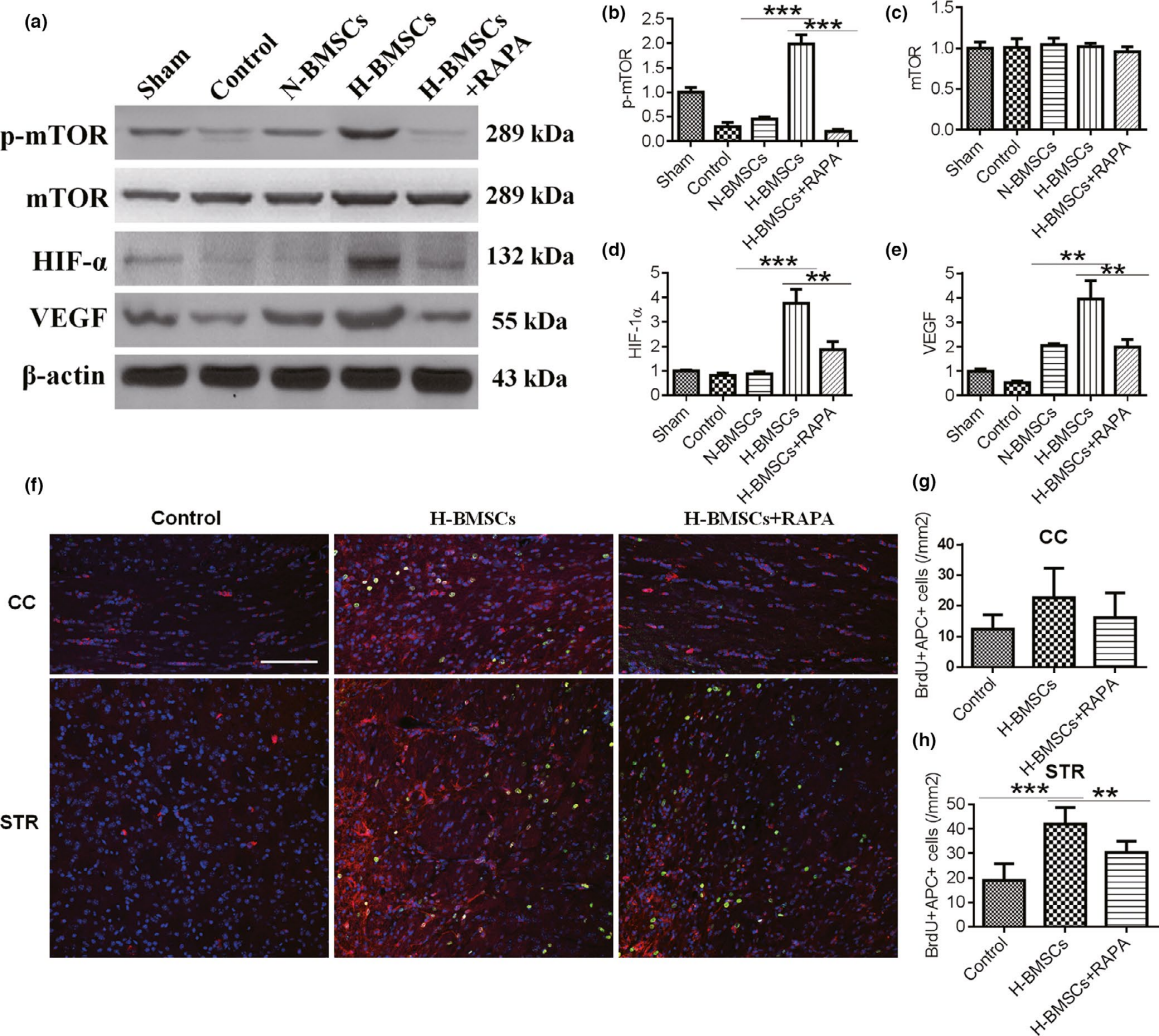
H‐BMSC transplantation prevented inhibition of the mechanistic target of rapamycin (mTOR) pathway in the brain after TBI. (a) Western blot analysis of phosphorylated (p)‐mTOR, mTOR, hypoxia‐inducible factor‐1a (HIF‐1α), and vascular endothelial growth factor (VEGF) expression in the brain 2 days after BMSC transplantation. Rapamycin (RAPA) was dissolved in DMSO and added to H‐BMSCs immediately at a concentration of 5 μg/ml before hypoxic treatment. DMSO alone was used as a diluent control in the N‐BMSC and H‐BMSC groups. (b–e) p‐mTOR, mTOR, HIF‐1α, and VEGF intensity levels were measured and calculated as fold changes over sham. (f) The CC and STR in the brain 35 days after TBI were double‐labeled with antibodies against APC (red) and BrdU (green). RAPA was dissolved in DMSO and immediately added to H‐BMSCs at a concentration of 5 μg/ml before hypoxic treatment. The overlay images are shown. The scale bars represent 100 μm. (g) Pooled data showing the effect of BMSC treatment on the number of APC^+^/BrdU‐positive cells in the CC 35 days after TBI. One‐way ANOVA showed a significant effect of H‐BMSC compared with the control treatment (*n* = 6 mice). (h) Pooled data showing the effect of BMSC treatment on the number of APC^+^/BrdU‐positive cells in the STR 35 days after TBI. One‐way ANOVA showed a significant effect of H‐BMSC compared with the control treatment (*n* = 6 mice). These sections were costained with DAPI (blue) to reveal nuclear morphology. Data are all presented as the mean ± *SD*. **p < .01, ***p < .001

HIF‐1α is reportedly activated by mTOR (Land & Tee, 2007) and has been further proven to inhibit neuronal apoptosis in rat brains after hypoxic‐ischemic injury (X. Wang, Li, Wu, Bu, & Qiao, 2016). In addition, mTOR has also been reported to be involved in TBI in mice (Ding et al., 2015; Nikolaeva, Crowell, Valenziano, Meaney, & D'Arcangelo, 2016; Zhu et al., 2014). Thus, the potential roles of HIF‐1α and its regulator mTOR as well as the HIF‐1 target gene VEGF in the protective effects of BMSC transplantation in TBI were explored. In the control group, CCI demonstrated a significant decrease in p‐mTOR 2 days after BMSC transplantation compared with the expression in the sham group (Figure 7a,b), and no significant change in total mTOR levels (Figure 7c). In addition, parallel reductions in HIF‐1α and VEGF were observed (Figure 7d,e) in both the control and N‐BMSC groups. However, the levels of p‐mTOR, HIF‐1α, and VEGF returned to sham levels after H‐BMSC transplantation. These results suggest that the mTOR pathway may be involved in the protective effect of H‐BMSCs in TBI. Therefore, we tested the effects of an inhibitor of the mTOR pathway (RAPA) in conjunction with H‐BMSCs. As expected, RAPA effectively decreased p‐mTOR levels (Figure 7a,b), suggesting that pre‐injections with RAPA could inhibit the protective effect of H‐BMSCs in TBI.

To investigate the proliferation in the CC and STR with rapamycin pretreatment in TBI, we further performed coimmunostaining for BrdU and APC in the CC and STR from the control and H‐BMSC–treated animals with or without RAPA pretreatment (Figure 7f–h). As expected, more BrdU/APC‐positive cells were observed in the H‐BMSC–treated group than in the control and N‐BMSC groups both in the CC (Figure 7f,g) and the STR (Figure 7f,h). However, when the mice were pretreated with RAPA, proliferation was inhibited, as red fluorescence was lower in these mice than in the H‐BMSC–treated mice.

## 4 DISCUSSION

Traumatic brain injury is a trauma‐induced brain disorder from which a growing number of young adults are suffering from without available effective therapeutic agents (Dixon, 2017). However, BMSC transplantation is regarded as having potential therapeutic benefits for TBI (Shen et al., 2016). After transplantation, BMSCs differentiate into neurons and were reportedly able to facilitate the regeneration of injured axons and promote functional recovery after injuries to the central nervous system (Carbonara et al., 2018; Neirinckx et al., 2015; Shen et al., 2016). Unfortunately, due to poor survival environment for engrafted cells within the lesion sites restrained the performance of this cell‐based therapy (Wang, Zhou, et al., 2015). Hypoxic preconditioning (HP) has been reported to increase ischemic tolerance, reduce apoptosis, and cause gene expression alterations (Feng & Bhatt, 2015; Huang et al., 2013). Our previous studies confirmed that pretreatment of BMSCs with hypoxia (1% O_2_) at every passage for 8 hr could effectively improve the effects of cell‐based therapies after ischemic stroke (Chen et al., 2017). Therefore, in this study, we also used 8 hr of HP for BMSC cell therapy after TBI. Our investigation was focused on whether H‐BMSC transplantation was able to result in greater therapeutic effects than N‐BMSC transplantation in TBI. Here, H‐BMSC transplantation was shown to decrease the cortical tissue loss significantly more than N‐BMSC transplantation. We further demonstrated that H‐BMSCs ameliorated sensorimotor function and cognitive deficits, and promoted white matter repair and axonal remyelination up to 35 days after TBI. Taken together with previously reported findings, this study supports the view that transplantation of HP‐treated BMSCs is a suitable therapeutic candidate for trauma‐induced cell death in both gray matter and white matter.

The complications after TBI cannot be attributed to the initial tissue damage only. Post‐TBI secondary events such as release of proteases, free radical formation, and excitotoxicity can result in demyelination, axonal degeneration, neuronal death, cavitation, and glial scarring around the area of initial damage (Wang et al., 2011, 2013). On the basis of the capacity of BMSCs for self‐renewal and differentiation into target cells (Guo et al., 2010), we detected the demyelination and regeneration effects in the CC and STR using immunofluorescence analysis. H‐BMSC treatment for 5 weeks turned out to induce the expression of MBP and NF200 both in the CC and the STR. Furthermore, compared to N‐BMSC grafts, H‐BMSC grafts resulted in a sharp increase in the number of NG2‐positive fibers and APC‐positive varicosities both in the CC and STR. The underlying mechanisms involved in this process could be associated with remyelination and neurogenesis. The results, therefore, provide further evidence to support the beneficial potential of H‐BMSC transplantation as an option of TBI treatment.

Although we found that H‐BMSCs were the most effective therapy for TBI, the underlying mechanisms involved in the therapeutic effects of HP‐treated BMSCs for TBI are still unknown. To elucidate the potential mechanisms underlying the protective effects of H‐BMSCs in TBI, we assessed the levels of p‐mTOR, HIF‐1α, and VEGF before transplantation with cultured BMSCs. The results showed that HP enhanced the expressions of p‐mTOR, HIF‐1α, and VEGF. BMSCs are consisted of stem cells and progenitor cells, which are able to produce a series of growth factors that facilitate neuronal survival and axonal growth (Buzoianu‐Anguiano et al., 2015; Zhang et al., 2018). Among these factors, VEGF can induce angiogenesis. We further proved that H‐BMSC transplantation can promote an increase in APC‐positive cells in both the CC and the STR by increasing the activity of the mTOR/HIF‐1α/VEGF pathway in the presence of RAPA. The underlying mechanisms may involve increased expressions of HIF‐1α and VEGF, which promote angiogenesis and neurogenesis, reduce neuronal death, and facilitate neuron function recovery. Therefore, H‐BMSCs should be useful in the reversal of neuronal fate under injured conditions. These results suggest that H‐BMSC treatment may crucially affect neural remodeling after injury. In addition, HP enhanced the therapeutic efficacy of BMSCs in the TBI model. However, the present study still has some limitations. Results obtained from animal experiments may not be directly applicable to humans; therefore, more comprehensive studies are required on this subject in the future.

## 5 CONCLUSION

In conclusion, H‐BMSC transplantation not only ameliorated neurological deficits, but also enhanced axonal regeneration and remyelination in mice after TBI, which may involve the mTOR/HIF‐1α/VEGF pathway. This study provided novel biological and histopathological evidences for TBI treatment that H‐BMSC transplantation may serve as a promising therapeutic option for both gray matter and white matter.

## CONFLICT OF INTEREST

The authors declare no conflicts of interest.

## AUTHORS’ CONTRIBUTION

G.H.W. conceived, organized, and supervised the study; L.H.S., X.Y.Y., and D.Q.G performed the experiments; Q.Q.L., J.C., L.Z.D., and Y.C.S contributed to the analysis of data; Q.Q.L. and G.H.W. prepared, wrote, and revised the manuscript.

## Data Availability

The data that support the findings of this study are available from the corresponding author upon reasonable request.
